# Global seasonal Sentinel-1 interferometric coherence and backscatter data set

**DOI:** 10.1038/s41597-022-01189-6

**Published:** 2022-03-11

**Authors:** Josef Kellndorfer, Oliver Cartus, Marco Lavalle, Christophe Magnard, Pietro Milillo, Shadi Oveisgharan, Batu Osmanoglu, Paul A. Rosen, Urs Wegmüller

**Affiliations:** 1 Earth Big Data LLC, P.O. Box 114, Woods Hole, MA 02543 USA; 2 GAMMA Remote Sensing AG, Worbstr. 225, 3073 Gümligen, Switzerland; 3 Jet Propulsion Laboratory, California Institute of Technology, 4800 Oak Grove Drive, Pasadena, CA 91109 USA; 4 University of Houston, Cullen College of Engineering, 5000 Gulf Freeway, Houston, TX 77004 USA; 5 NASA/GSFC, 8800 Greenbelt Road, Greenbelt, MD 20771 USA

**Keywords:** Ecology, Environmental sciences, Solid Earth sciences

## Abstract

This data set is the first-of-its-kind spatial representation of multi-seasonal, global C-band Synthetic Aperture Radar (SAR) interferometric repeat-pass coherence and backscatter signatures. Coverage comprises land masses and ice sheets from 82° Northern to 79° Southern latitudes. The data set is derived from multi-temporal repeat-pass interferometric processing of about 205,000 Sentinel-1 C-band SAR images acquired in Interferometric Wide-Swath Mode from 1-Dec-2019 to 30-Nov-2020. The data set encompasses three sets of seasonal (December-February, March-May, June-August, September-November) metrics produced with a pixel spacing of three arcseconds: 1) Median 6-, 12-, 18-, 24-, 36-, and 48-days repeat-pass coherence at VV or HH polarizations, 2) Mean radiometrically terrain corrected backscatter (γ^0^) at VV and VH, or HH and HV polarizations, and 3) Estimated parameters of an exponential coherence decay model. The data set has been produced to obtain global, spatially detailed information on how decorrelation affects interferometric measurements of surface displacement and is rich in spatial and temporal information for a variety of mapping applications.

## Abstract (summary)

**Table:** 

Measurement(s)	Seasonal C-band SAR coherence and backscatter from Dec. 2019 to Nov. 2020
Technology Type(s)	SAR interferometric and radiometric terrain correction processing of Sentinel-1 SAR data
Sample Characteristic - Environment	Global land an coastal sea ice surfaces
Sample Characteristic - Location	Earth from 82 degrees northern to 79 degrees southern latitude

## Background & Summary

Interferometric Synthetic Aperture Radar (InSAR) measurements of surface deformation and change provide an important tool for understanding the dynamics of earthquakes, volcanoes, landslides, glaciers, groundwater variation, mantle processes, and ecological processes in agriculture, wetland, and vegetation disturbances, among other applications. InSAR processing consists of cross-correlating two complex SAR images of the same terrain and is routinely used to measure millimetre-level surface deformation by making observations on temporally separated images.

The accuracy of InSAR measurements is influenced by ionospheric and tropospheric propagation delays, as well as the interferometric coherence, synonymously referred to as interferometric correlation. The contribution of decorrelation, i.e., loss of coherence, to the deformation measurement error $\left({\sigma }_{{d}_{los}}\right)$ along the radar line-of-sight for a single interferogram can be approximated with:

1 $${\sigma }_{{d}_{los}}=\frac{\lambda }{4\pi }\frac{1}{\sqrt{2{N}_{L}}}\sqrt{\frac{1-{\gamma }_{eff}^{2}}{{\gamma }_{eff}^{2}}}$$

where *N*_*L*_ is the effective number of SAR looks, *γ*_*eff*_ the effective coherence between two SAR images, and *λ* the signal wavelength. Provided appropriate image formation and interferometric processing, the interferometric coherence observed over a target of interest depends on the length of the interferometric baseline, system noise, and temporal decorrelation^1–4^

2 $${\gamma }_{eff}={\gamma }_{baseline}\times {\gamma }_{noise}\times {\gamma }_{temporal}$$

and thus on i) technical specifications of the interferometric radar constellation such as signal wavelength, interval between consecutive acquisitions, or the orbital tube^5^ and ii) the temporal stability of geometric and dielectric properties of the observed target. To assess the performance of planned satellite missions, NASA JPL developed a Science Performance Model for the upcoming NISAR mission^6^ to simulate the line-of-sight error associated with mission plan. This model has been extended to support trade studies on NISAR continuity measurements under NASA’s Surface Deformation and Change (SDC) Architecture study^7^, which includes constellation concepts at different wavelengths and spacecraft constellation configurations. Production of the data set described in this paper was motivated by the need to obtain spatially detailed information on how decorrelation at different repeat intervals affects line-of-sight displacement measurements at C-band (5 GHz), and to use this as a reference in modelling similar decorrelation rates at other wavelengths^8^.

With the goal to estimate seasonal variations of repeat-pass coherence at different repeat intervals, this data set was developed by interferometric processing of all Sentinel-1 SAR data acquired over the course of one year between the 1^st^ of December 2019 and the 30^th^ of November 2020. This constituted a volume of about 205,000 Sentinel-1 Single-Look-Complex (SLC) scenes acquired in Interferometric Wide-Swath mode^9^ with a raw data volume of about 0.9 Petabytes. Each possible image combination with repeat intervals up to 48 days was considered as input into estimates of seasonal median coherence at 6-, 12-, 18-, 24-, 36- and 48-days repeat intervals of Sentinel-1’s co-polarized observations (VV and HH polarizations). For additional value in interpreting global seasonal SAR measurement patterns, seasonal means of backscatter amplitudes for all co- and cross-polarized observations (VV, VH, HH, HV) were generated, thus complementing recently published maps of mean annual Sentinel-1 backscatter at 10 m spatial sampling with information on seasonal variations in backscatter^10^. As ancillary data, local incidence angle and layover and shadow maps were produced for all 175 Sentinel-1 imaging swaths. Finally, a coherence decay model was fitted to the repeat-pass coherence measurement as function of the repeat interval in each season which provided two seasonal model fit parameters per season describing the long-term coherence and coherence decay rates.

The primary motivation for producing this rich data set was to support mission design and application development in the context of measuring surface deformation. To this end, numerous previous studies have demonstrated the value of C-band interferometric coherence and backscatter time series for various applications, e.g., the mapping of land cover^11–13^, biophysical parameters of forests^14–23^ or crops^18,24,25^, soil moisture^26,27^, sea ice, icesheets and glaciers^28–30^, or properties of snow^31–33^. This is the first globally consistent data set supporting such applications at regional to global scales.

## Methods

### Sentinel-1 data selection

The Copernicus Sentinel-1 mission was launched by the European Space Agency (ESA) in 2014 with the Sentinel-1A satellite, complemented with the second Sentinel-1B satellite in 2016. Each satellite has a 12-days repeat cycle. Continuity of the Sentinel-1 mission has been approved by ESA until 2030 and replacement satellites will be launched. The satellites operate in different acquisition modes over different parts of the globe. Land masses are covered primarily by the Interferometric Wide-Swath Mode (IW) with a 250 km swath width across-track. Single-look-complex (SLC) Level 1.1 data are required for interferometric processing. Along-track, Sentinel-1 data are sliced into consecutive frames (slices) of about 250 km length. Data are distributed via ESA’s Scientific Sentinel-1 Hub, which is mirrored at NASA’s Alaska Satellite Facility DAAC (ASF-DAAC). During production, Sentinel-1 SLC data were accessed on the ASF-DAAC data repository which resides on Amazon’s AWS S3 bucket in region us-west-2.

**Fig. 1:**
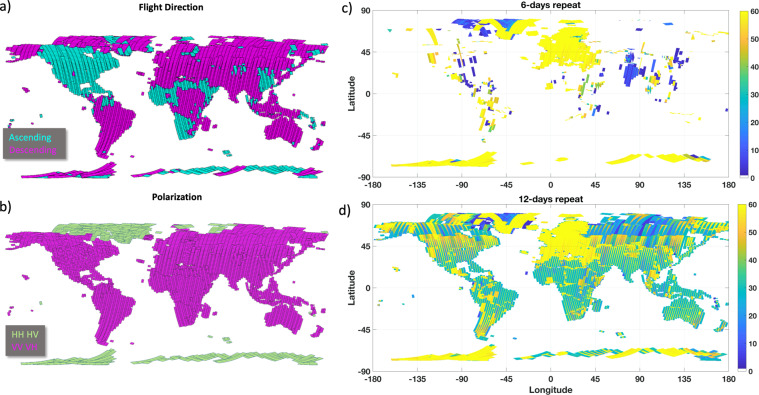
Flight direction, polarization mode, and InSAR stack sizes of 6- and 12-days repeat coverage of Sentinel-1 data acquired between December 1^st^ 2019 and November 30^th^ 2020 selected for processing.

Sentinel-1 satellites cover various parts of Earth in ascending and descending flight direction in a total of 175 relative orbits. ESA’s flight plan has some areas covered every six days and in both flight directions, predominantly over Europe. For the production of this data set, Sentinel-1 SLC frames were selected from all available scenes acquired between December 1^st^ 2019 and November 30^th^ 2020. Over the one-year timeframe, a maximum of 30 to 31 acquisitions at 12-days repeat, and 60 to 61 acquisitions at 6-days repeat intervals can be expected. The following selection criteria were applied consecutively to achieve global coverage with uniform distribution of acquisitions across seasons (Fig. 1):

- Global descending data (Fig. 1a) were selected where the one-year stack size had at least 25 acquisitions.
- Spatial gaps were filled with ascending data (Fig. 1a) where the one-year stack size had at least 25 acquisitions.
- For spatial consistency, over conterminous North America north of Panama, preference was given to ascending data where both ascending and descending data existed with stack sizes over 25 acquisitions.
- For stack sizes less than 25 acquisitions, preference was given to the flight direction with the larger number of acquisitions.
- Remaining gaps were filled with data from the flight direction available.

Arctic and Antarctic regions are typically covered with polarization modes of horizontal transmit (HH single- or HH/HV dual-polarization). Figure 1b shows the global distribution of the processed data in horizontal and vertical polarization transmit modes, respectively. Table 1 summarizes the number of selected scenes in the two flight directions and various polarization modes. The total number of processed Sentinel-1 SLC frames came to ~205,000 scenes with a total raw input data volume of about 850 Terabytes. Figure 1c,d show the spatial distribution of the final scene selection with the number of 6- and 12-days repeat-pass image pairs. Consistent 6-days repeat coverage with about sixty image pairs from either ascending or descending orbits could be processed over Europe, the coastal areas of Greenland and Antarctica, and some smaller areas around the world; note that in some regions (e.g., India, interior Greenland, Northern Canada, Eastern China) 6-days repeat coverage was available in certain seasons only (Fig. 1c). A consistent coverage with 12-days repeat-pass imagery, instead, could be processed almost globally with the nominal maximum of about thirty repeat-pass pairs in areas where only one satellite, Sentinel-1A or Sentinel-1B, acquired data in all but few areas above 60° N in Canada, Greenland, or Russia (Fig. 1d). In some small areas in the Midwestern United States, the Khabarovsk region in Far-Eastern Russia, or in the Northern Sahara, neither Sentinel-1A nor Sentinel-1B acquire data in IW mode, leading to small gaps in the final data set.

**Table 1:** Number of Sentinel-1 Single Look Complex scenes processed.

Flight Direction	Polarization Mode	Scene Count
ASCENDING	HH	5,849
ASCENDING	HH + HV	2,119
ASCENDING	VV + VH	55,697
DESCENDING	HH	6,373
DESCENDING	HH + HV	7,059
DESCENDING	VV	87
DESCENDING	VV + VH	127,498

### Processing approach

**Fig. 2:**
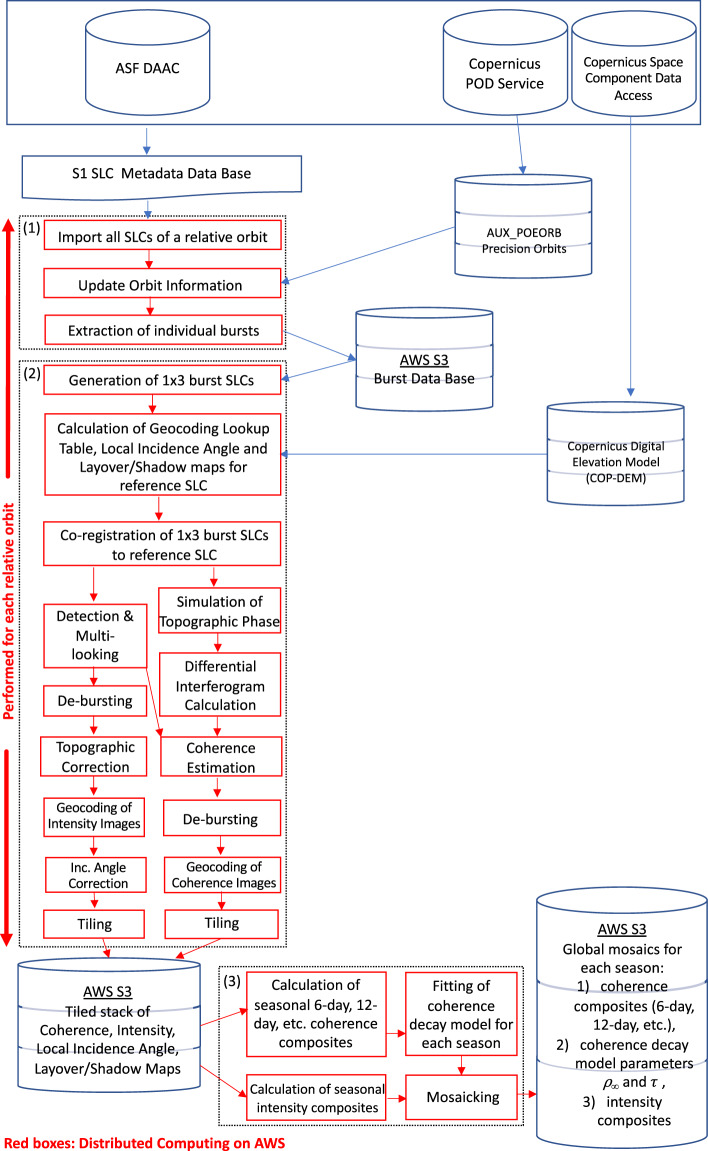
Implementation of the Sentinel-1 interferometric processor in the AWS cloud environment.

The overall processing workflow was developed based on the interferometric processing software developed by GAMMA Remote Sensing and geared towards efficient processing in the Amazon Web Services (AWS) cloud environment utilizing Earth Big Data LLC’s cloud scaling solutions. The workflow is divided into three main blocks as illustrated in Fig. 2. Sentinel-1A and -1B acquire data along 175 relative orbits/orbital tracks. Blocks 1 and 2 were processed on a per relative orbit basis; block 3 was initiated after blocks 1 and 2 had been completed for all relative orbits.

#### Processing Block 1

For each SLC of a given relative orbit, processing block 1 entailed:

1. Conversion of SLC image files to a GAMMA specific format. Each Sentinel-1 SLC, covering an area of ~250 × 250 km, consists of six SLC image files (one SLC image file for each of the three sub-swaths in co- (VV or HH) and cross-polarizations (VH or HV).
2. Compensation of the SLC amplitudes for the noise equivalent sigma zero (NESZ).
3. The orbit state vectors provided with the original Sentinel-1 SLCs were updated with the precision state vectors (AUX_POEORB) distributed by the Sentinel-1 payload data ground segment 20 days after data take with a precision (3σ) generally of the order of 1 cm (target requirement < 5 cm).
4. Each Sentinel-1 sub-swath SLC typically comprises nine to ten (sometimes more) individual bursts separated by a few no-data lines, a format that we will from now on refer to simply as “burst SLC”. For each Sentinel-1 burst a unique burst ID number was defined that permits identifying the corresponding bursts in all repeat acquisitions. We then extracted individual bursts in each sub-swath and wrote each individual burst image and associated parameter files to a temporary AWS S3 bucket.

#### Processing Block 2

Once all SLCs of a given relative orbit have been imported and individual bursts have been stored to AWS S3, the pre-processing block for generating terrain-corrected and geocoded coherence and backscatter images was initiated. The processing block started with grouping all bursts in a selected relative orbit into 1 × 3 burst groups/segments (3 sub-swaths comprising 1 burst each) and the creation of consistent stacks of burst SLCs for each 1 × 3 burst segment across all multi-temporal Sentinel-1 observations of the same relative orbit per segment. All processing steps described below were then performed on a segment-by-segment basis. The main processing parameters have been summarized in Table 2.

**Table 2:** Processing and product target parameters.

Processing Parameter	Value
Multi-looking factors in range and azimuth for interferogram generation	12 × 3 in range and azimuth
Multi-looking factors in range and azimuth for backscatter intensities	36 × 9 in range and azimuth
Adaptive coherence estimation window size	3 × 3 to 9 × 9 pixels
Spatial Reference System	Equiangular, EPSG:4326
Pixel posting in longitude and latitude	0.00083333333° × 0.00083333333°
Coherence bias	<0.05
Backscatter Equivalent Number of Looks	>100
Digital Elevation Model	COPDEM-90

Having created consistent stacks of burst SLCs for all repeated observations from the same relative orbit, we defined for each burst SLC segment a reference (primary) acquisition to which all other (secondary) burst SLCs of the same segment were co-registered. In the next step, transformation functions (so called geocoding look-up tables) for resampling between slant-range and map geometry were calculated based on the available orbit information and the 3 arcsecond Copernicus DEM^34^ for the burst SLC segment selected as reference. Alongside the geocoding look-up table, maps of the local incidence angle and layover/shadow masks were produced. An intensity cross-correlation method was subsequently applied to check the geometric accuracy of the transformation functions^35^. This accuracy check uses a simulated SAR backscatter image, calculated by means of the DEM and an assumed topography dependence of the backscatter^36^, as reference geometry against which the matching of the Sentinel-1 intensity images in small image chips was performed. The standard deviation of offsets in range and azimuth with respect to a polynomial regression fit to the offset estimates served as quality control. Throughout the entire processing chain, the geocoding look-up table served two purposes:

1. Resampling of the DEM in map projection (geographic, EPSG: 4326) to the slant-range geometry of the reference SLC image to be able to i) consider topography related offsets between reference and secondary SLCs in the co-registration step, and ii) simulate the topographic phase when computing the differential interferograms,
2. terrain-corrected geocoding of all imagery that matches the geometry of the selected reference acquisition/segment (i.e., all coherence and backscatter intensity images derived from SLCs that were co-registered to the selected reference).

The co-registration of the burst SLCs was done based on transformation functions (referred to as co-registration look-up tables) derived from the orbit information and the DEM resampled to the slant-range geometry of the selected reference burst SLC. The accuracy of the orbit information available for Sentinel-1 suggests that these transformation functions allow for resampling the secondary burst SLCs to the geometry of the reference with a co-registration accuracy of few hundredth of a pixel, i.e., sufficient to preserve coherence^5^. However, to ensure accurate and reliable co-registration, we also applied a single matching refinement (intensity cross-correlation) that served, if necessary, as correction of the co-registration look-up tables and to document the quality of the co-registration.

For simulating the baseline-dependent interferometric phase introduced by topography, the DEM in slant-range geometry and an orbit-based phase model was used. In the calculation of the differential interferometric phase (in SAR geometry), the topographic phase was subtracted and common band filtering in range was applied. Differential interferograms were calculated for all 6-, 12-, 18-, 24-, 36-, and 48-days repeat-pass image pairs in the co-registered stack of images. This was done in a multi-look burst geometry, meaning that bursts were kept separate so that no data from different bursts were combined in the estimation. The differential interferograms were calculated using 12 range and 3 azimuth looks resulting in interferograms with a ground-range resolution of about 40 m. The number of interferograms varied dependent on whether 6- or 12-days repeat-pass imagery was available. In case of a complete 6-days coverage throughout the observation period (Fig. 1c), a total of the order of 340 interferograms could be computed. In case of a consistent 12-days coverage (Fig. 1d), the number of interferograms was reduced to about 110.

An adaptive coherence estimator was used to produce coherence maps from all differential interferograms in multi-look burst geometry. The adaptive moving window estimator derives an initial estimate using a fixed 3 × 3 pixels estimation window size. Based on the initial estimate, a spatially adaptive estimator was applied so that the estimation included 5 × 5 or 7 × 7 pixels in multi-look geometry for low coherence levels. In the final coherence estimation, inverts of the deviations of the 3 × 3 pixels coherence estimates located within the larger estimation window served as weights to preserve spatial detail in the final estimate. Given the multi-looking and the size of the adaptive estimation window, coherence was estimated with a nominal number of looks of at least 324 and up to 1764. The large number of looks ensured a minimization of the bias inherent to coherence estimation^2^. In areas for which decorrelation is expected to be complete (e.g., water surfaces, layover), the average coherence was of the order of 0.03. Finally, to produce seamless coherence images in slant-range geometry without no-data lines between subsequent bursts, coherence estimates for the three bursts per segment were mosaicked (referred to as de-bursting in ESA terminology). Only one of the available estimates in regions of overlapping bursts/sub-swaths was used. Averaging these would have resulted in different statistics between areas with or without burst overlap. All coherence images were eventually geocoded to the required map geometry with a 3 arcsecond sampling using the geocoding look-up table calculated for the reference acquisition.

Coherences were processed at co-polarization only (VV or HH dependent on data availability). The processing of coherence for cross-polarization data was not in the scope of the project. In the case of backscatter intensity, both polarizations acquired by Sentinel-1 in IW mode (VV/VH or HH/HV) were instead considered. Alongside the coherence estimation, we radiometrically terrain corrected (RTC) backscatter images acquired at co- and cross- polarizations. The pre-processing of backscatter intensity images deviated from the processing of coherence following the co-registration. The co- and cross-polarization burst SLCs were detected and multi-looked with multi-looking factors of 36 in range and 9 in azimuth. The multi-look backscatter images in burst geometry (calibrated to σ^0^) were subsequently de-bursted to create seamless images in slant-range geometry combining bursts from all three sub-swaths. In order to calibrate the backscatter images over sloped terrain, the varying extent of the ground area covered by image pixels dependent on topography was calculated as with Frey *et al*.^36^. For each image, a normalization factor for the topographic correction of backscatter was calculated with the ratio of the pixel area referring to flat terrain (ellipsoid referenced *σ*^*0*^ areas) and the “true” pixel area considering topography (*γ*^*0*^ normalization area to produce “terrain-flattened” *γ*^*0*^ backscatter images^37^. All backscatter images in co- and cross-polarizations were geocoded using the same geocoding look-up table used for geocoding the coherence imagery.

All coherence, backscatter, and ancillary imagery (local incidence angle maps, layover/shadow maps) were fully pre-processed for all 1 × 3 burst segments in a relative orbit, all resulting images were tiled to a fixed global 1° × 1° grid and stored as GeoTIFF files in a temporary AWS S3 storage bucket together with quality reports generated during processing. The quality/ancillary reports associated with each image comprised information on i) perpendicular baselines, ii) quality indicators for the co-registration, and iii) quality indicators for the geocoding accuracy.

#### Processing Block 3

Having pre-processed all coherence and backscatter imagery and created a complete global tile data base, the final processing block comprised i) seasonal compositing of coherence and intensity imagery, ii) fitting of a coherence decay model to seasonal stacks of coherence at pixel level, and iii) creation of global mosaics.

For each tile, seasonal composites of the coherence at different repeat intervals and backscatter imagery were calculated. We calculated the median coherence based on all coherence estimates per tile of a given repeat interval (6, 12, 18, 24, 36, and 48) per three-month period: 1) December, January, February 2) March, April, May 3) June, July, August, and 4) September, October, November. We chose the median operation to account for outliers. In the case of the backscatter intensity products, we calculated per three-month period the average backscatter intensity in VV and VH, or HH and HV, polarization.

The decay of coherence with increasing repeat interval was modelled for each season at pixel-level with the exponential model^38^

3 $${\gamma }_{t}\left(t\right)=\left(1-{\rho }_{\infty }\right){e}^{-t/\tau }+{\rho }_{\infty }$$

where *ρ*_*∞*_ and *τ* denote the long-term coherence and rate of coherence decay with increasing repeat interval, respectively. The estimation of the coherence model parameters *ρ*_*∞*_ and *τ* was done based on the median-aggregated seasonal coherence estimates. The pixel-level estimates for *ρ*_*∞*_ and *τ*, together with a quality indicator of the model fit, i.e., root mean square difference between modelled coherence and individual non-aggregated coherence observations, were stored in form of images in the same geometry as the tiled Sentinel-1 datasets. Curve fitting was achieved with the standard Levenberg-Marquardt least-squares regression unless the initial model fit resulted in non-physical solutions for the model parameters, i.e., negative estimates for *ρ*_*∞*_ (cf., Technical Validation). In such cases, we adopted a different regression technique with the Trust Region Reflective regression^39^, which is significantly slower (and thus not applied globally by default) but allows for constraining the potential range of parameter estimates, i.e., 0 ≤ρ_∞_≤1.

Finally, we created global mosaics of each layer, i.e., seasonal 6-, 12-, 18-, 24-, 36-, and 48-days coherence at VV- and HH polarizations, seasonal backscatter in co- (VV and HH) and cross-polarizations (VH and HV), as well as ρ_∞_, τ, and the corresponding model RMSE of the model fit (see Data Records).

## Data Records

The data set is available in three main data records, detailed in the following sections:

1. 1 × 1-degree tiles. Each tile contains GeoTIFFs at 3 arcsec pixel spacing of all metrics available in the tile.
2. Global Virtual Raster Tables (VRT) with external overview files of seasonal backscatter, coherence, rho, tau, and rmse including VRT stacks of seasons.
3. Global mosaicked tiles as cloud optimized GeoTIFFs at 0.01-degree pixel spacing for each of the computed metrics.

The data records are stored and openly accessible at the following locations:

1. NASA’s Alaska Satellite Facility DAAC (ASF-DAAC): Permanent record with open access for users with the freely obtainable Earthdata login. URL^40^: https://asf.alaska.edu/datasets/derived/global-seasonal-sentinel-1-interferometric-coherence-and-backscatter-dataset/
2. Open and anonymous access at Amazon Web Services Registry of Open Data with descriptors and tutorials: https://registry.opendata.aws/ebd-sentinel-1-global-coherence-backscatter/

**Fig. 3:**
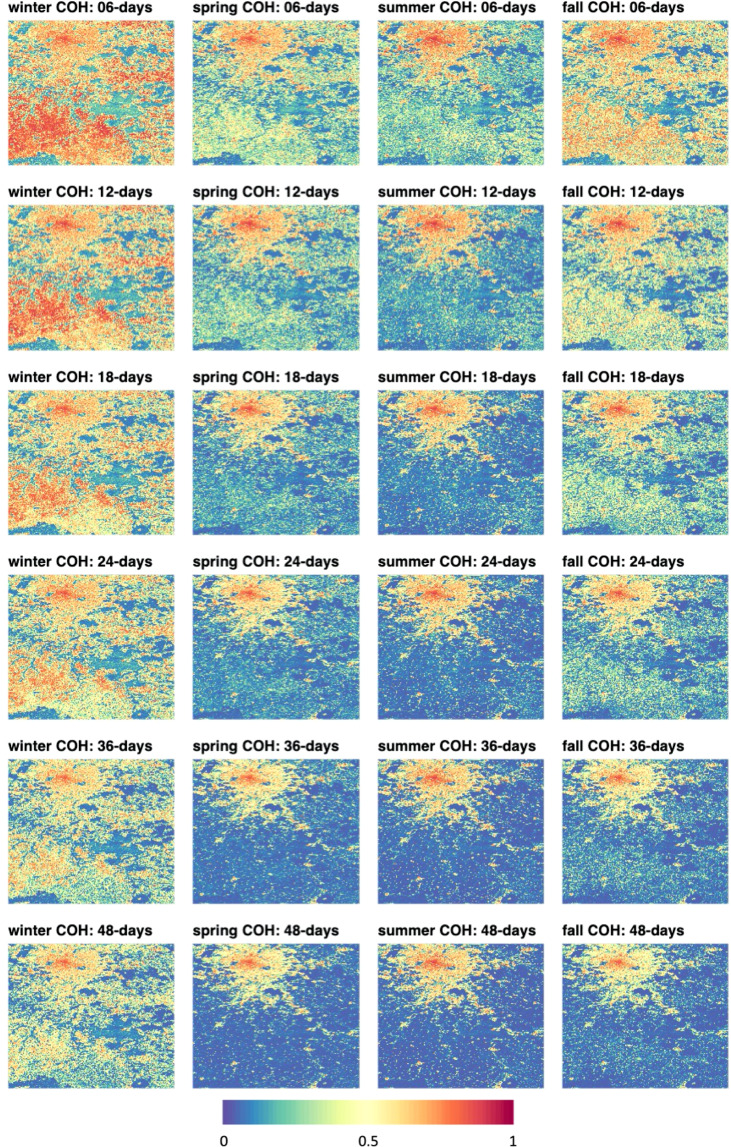
Tile N49E002 showing Paris, France in the upper left. Seasonal coherence. Columns show seasons, rows show median seasonal coherences of 6-, 12-, 18-, 24-, 36-, and 48-days repeat. Color scale show coherence values from 0 to 1.

The 1 × 1-degree tiles of the data set form the basic units of produced image data. All computed metrics are stored as image files in GeoTIFF format where the extent of the images covers exactly 1 degree in longitude and 1 degree in latitude with the origin of the upper left pixel at a full degree in latitude and longitude respectively. All GeoTIFFs have 3 arcseconds resolution in both dimensions and are output to a geographic reference system based on the WGS84 ellipsoid (EPSG:4326). Thus, each file in a tile has a raster dimension of 1,200 pixels by 1,200 lines. For all images, no data values are coded as the digital number zero. Global coverage encompasses a total number of 25,334 tiles. For the tile covering the city of Paris, Fig. 3 exemplifies the seasonal coherence metrics. Table 3 summarizes the type of image products and data conversion to digital numbers. All images are stored in GeoTIFF format with lossless Lempel-Zip-Welsh (LZW) compression. Naming conventions for the GeoTIFF files are listed in Table 4.

**Table 3:** List of GeoTIFF image products and data conversion to digital numbers in a tile.

Metric		Format
Mean seasonal backscatter amplitudes γ^0^ (**AMP**)	Polarizations: HH & HV, VV & VH	Scaled to 16 bit unsigned integer with: DN = 10.0^((dB + 83.0)/20.0) where: dB = 10*log10(γ^0^)
Median seasonal 6,12,18,24,36,48-days repeat-pass coherences (**COHxx**)	Polarizations: HH, VV	Coherence ρ scaled to 8 bit unsigned integer with: DN = ρ*100
Seasonal coherence decay-model parameters ρ_∞_, τ, and RMSE (rho, tau, rmse)	Polarizations: HH, VV	ρ_∞_, τ, RMSE scaled to 16 bit unsigned integer with: DN = ρ_∞_/τ/RMSE*1000
Local incidence angle (**inc**)	Per relative orbit	DN = Degrees (8 bit unsigned integer)
Layover/shadow mask (**lsmap**)	Per relative orbit	8 bit unsigned integer where: 1 - No Shadow or Layover 5 - Layover 17 - Shadow 21 - Shadow in Layover

No data value for all products is the digital number zero (0).

**Table 4:** Naming conventions for tiled data set of seasonal coherences, backscatter, and coherence decay modelling results.

**ASF-DAAC resource URL:** https://sentinel1.asf.alaska.edu/global_coherence/s1/data/tiles/<TILEID>/<FILENAME> **AWS resource name:** arn:aws:s3:::sentinel-1-global-coherence-earthbigdata/data/tiles **AWS resource URL**: https://sentinel-1-global-coherence-earthbigdata.s3.us-west-2.amazonaws.com/data/tiles/<TILEID>/<FILENAME>
**Tile naming convention** Tile identifiers (TILEID) in the data set are labelled by the upper left coordinate of each 1×1 degree tile using North (N) and South (S), and East (E) and West (W) qualifiers. Tiles with the northern edge aligned with the equator are using N00 as the latitude identifier. Examples: • N48W090 covers the area of 47°–48° northern latitude and 90°–89° degrees western longitude • S01E012 covers the area of 1°–2° southern latitude and 12°–13° degrees eastern longitude • N00E000 covers the area of 0°–1° southern latitude and 0°–1° degrees eastern longitude
**File naming convention for backscatter, coherence, and model parameter files:** <TILEID>_<SEASON>_<POLARIZATION>_<METRIC>.tif with TILEID Identifier of tile referenced to upper left tile corner SEASON winter, spring, summer, or fall (referring to Northern hemisphere seasons) POLARIZATION vv, vh, hh, or hv METRIC AMP, COHxx, rho, tau, rmse with xx=06,12,18,24,36,48 Examples: • N49E012_winter_vv_COH06.tif • N49E012_spring_vv_COH48.tif • N49E012_summer_vh_AMP.tif
**File naming convention for incidence angle and layover/shadow files:** <TILEID>_<OOO><F>_<METRIC>.tif with TILEID Identifier of tile referenced to upper left tile corner OOO Relative Orbit Number from 001 to 175 F A or D for ascending or descending flight direction METRIC inc or lsmap Examples: • N49E012_095D_inc.tif

**Fig. 4:**
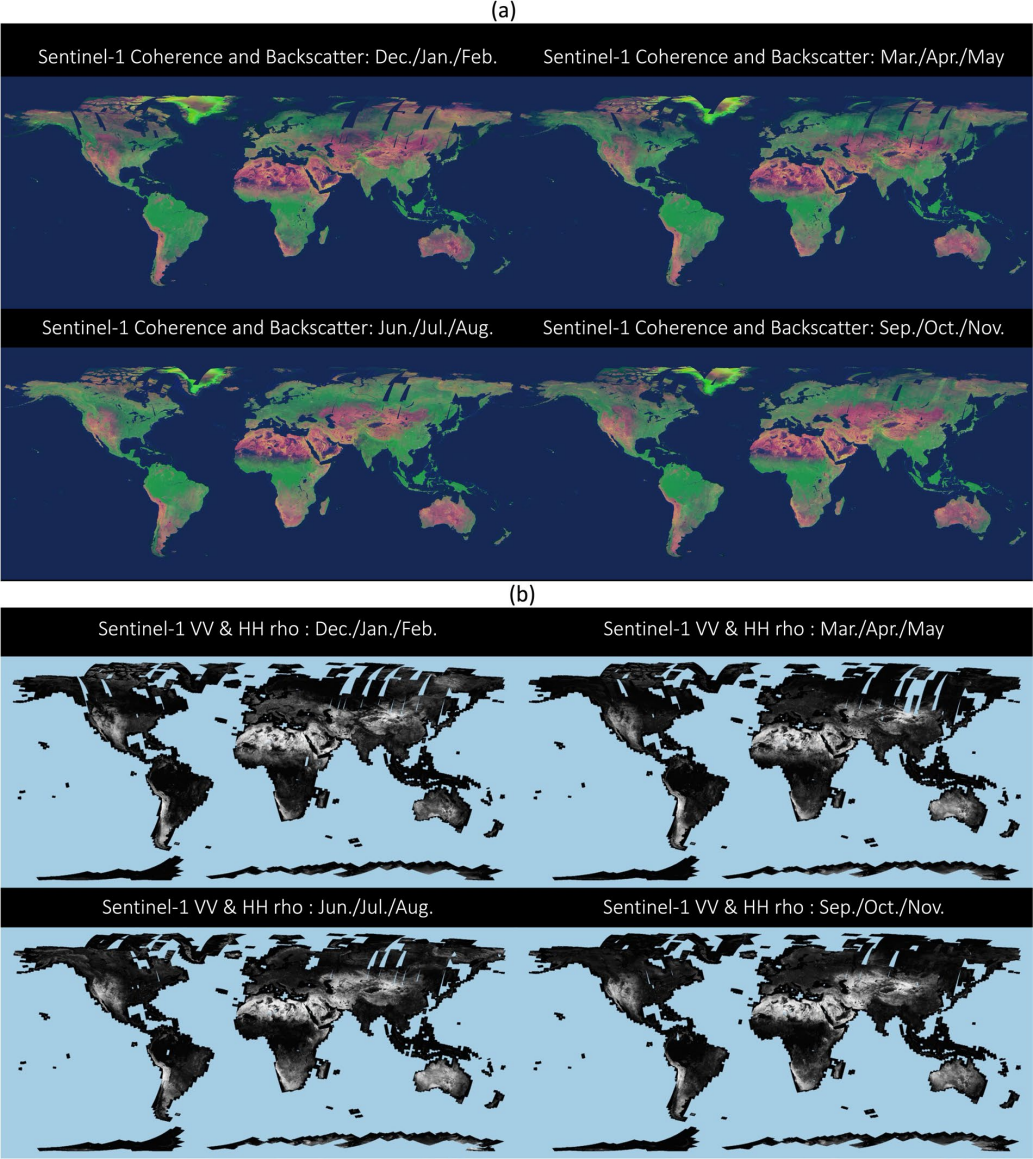
(**a**) Seasonal RGB mosaics of median VV 12-days coherence (red band), mean VH backscatter (green band), and mean VH/VV backscatter ratio (blue band). Colors are enhanced for visual effect, but not altered between seasons. (**b**) Seasonal mosaics of the coherence decay model parameter rho (ρ_∞_).

From all available tiles containing the various metrics, global Virtual Raster Tables (VRT) were generated with tools based on the Geospatial Data Abstraction Library (GDAL - https://gdal.org). VRTs are available for each seasonal mosaic of a given metric such as the 12-days coherence for the (northern hemisphere) summer or the mosaic of backscatter at VH polarization in winter. In addition, global VRTs are provided which contain all seasonal mosaics of a given metric. The naming of VRTs followed the naming convention described in Table 5. For visualization purposes, external overviews were produced on these files at four overview levels reducing the full resolution global mosaic by two-dimensional down-sampling with averaging by factors of 3 × 3, 9 × 9, 27 × 27, and 81 × 81. The external overview files are identified by having a filename identical to the global mosaics with the additional postfix “.ovr” appended. From the global VRTs described above, downsampled, cloud-optimized GeoTIFFs (COG) were generated with built-in overviews. Sample spacing in these mosaics are 0.01 degrees on both longitudinal and latitudinal directions. Figure 4 shows examples of these global mosaics as individual seasonal mosaics or in RGB colour combinations to enhance visual effects of patterns detected in the data records. The naming of the global mosaics followed the convention described in Table 6.

**Table 5:** Naming conventions for Virtual Raster Tables Files.

**ASF-DAAC resource URL:** https://sentinel1.asf.alaska.edu/global_coherence/s1/data/tiles/<VRTFILENAME > **AWS resource name prefix:** arn:aws:s3:::sentinel-1-global-coherence-earthbigdata/data/tiles/Global_ **AWS resource URL**: https://sentinel-1-global-coherence-earthbigdata.s3.us-west-2.amazonaws.com/data/tiles/<VRTFILENAME >
**File naming conventions for seasonal single metric VRTs:** Global_ < SEASON > _ < POLARIZATION > _ < METRIC > .vrt with SEASON winter, spring, summer, or fall (referring to Northern hemisphere seasons as proxy names for the months groupings of DJF, MAM, JJA, SON) POLARIZATION vv, vh, hh, or hv METRIC AMP, COHxx, rho, tau, or rmse with xx from 06,12,18,24,36,48 Examples: • Global_spring_vv_COH06.vrt • Global_winter_vh_AMP.vrt
**File naming conventions for all-season VRT stacks:** Global__<POLARIZATION>_<METRIC>.vrt with POLARIZATION vv, vh, hh, or hv METRIC AMP, COHxx, rho, tau, or rmse with xx from 06,12,18,24,36,48 Examples: • Global__vv_COH06.vrt • Global__hh_AMP.vrt

**Table 6:** Naming conventions for global GTiff mosaics with 0.01-degree sample spacing.

**ASF-DAAC resource URL:** https://sentinel1.asf.alaska.edu/global_coherence/s1/data/mosaics/<FILENAME> **AWS resource name:** arn:aws:s3:::sentinel-1-global-coherence-earthbigdata/data/mosaics/ **AWS resource URL**: https://sentinel-1-global-coherence-earthbigdata.s3.us-west-2.amazonaws.com/data/mosaics/<FILENAME>
**File naming conventions for global 0.01° sample spacing GeoTIFFs:** Global_<SEASON>_<POLARIZATION>_<METRIC>_100ppd.tif with SEASON winter, spring, summer, or fall (referring to Northern hemisphere seasons) POLARIZATION vv, vh, hh, or hv METRIC AMP, COHxx, rho, tau, or rmse with xx from 06,12,18,24,36,48 ppd pixels per degree Examples: • Global_spring_vv_COH06_100ppd.tif • Global_fall_vv_rho_100ppd.tif

## Technical Validation

The technical validation of the global coherence and backscatter products presented above aimed to (i) verify the processing quality of the products, and (ii) provide guidelines for the interpretation of the products with respect to the role of different decorrelation mechanisms. The processing quality of the coherence and backscatter products was evaluated with respect to the accuracy of geocoding and co-registration. To aid usage/interpretation of the coherence products and coherence decay modelling results, we provide an analysis of how different decorrelation mechanisms contributed to the coherence reported in the seasonal maps.

A prerequisite for differential interferometric processing is accurate geocoding lookup tables used to resample at the burst segment level i) all derived image products (coherence and backscatter imagery) from slant-range to the selected map geometry (i.e., the actual geocoding), and ii) the DEM from map geometry to slant-range to be able to simulate the topographic/flat earth phase and produce the differential interferogram. The precision orbit information available for Sentinel-1 and the Copernicus 3-arcsec DEM were used to calculate geocoding look-up tables for each acquisition (3-burst segment) selected as reference for the co-registration. The accuracy of the geocoding look-up tables was determined by means of cross-correlation of the multi-look intensity image (reference image) and an intensity image simulated from the DEM and resampled to slant-range geometry^36^. Since in areas without topography the lack of features in the simulated intensity image does not allow for estimating offsets, the offset statistics were evaluated on a per relative orbit basis. The offset statistics confirmed for all 175 relative orbits the high geolocational accuracy of the products with average offsets in azimuth and range of 2.1 m and 7.6 m, respectively.

One of the main challenges when interferometrically processing large volumes of Sentinel-1 SLC data is the accurate and reliable co-registration of SLCs. The co-registration was achieved by means of orbit models calculated from the precision orbit information and the DEM to account for topography in the case of non-zero baselines. A cross-correlation between the reference and the resampled dependent SLCs was applied for each 3-burst segment to verify the accuracy of the co-registration^35^. For each pair of images to be co-registered, the cross-correlation provided information about the residual offsets in range and azimuth. The offsets were generally within +/− 0.02 pixels in azimuth and mostly within +/− 0.1 pixels in range. The overall small offsets corroborate the high accuracy of the Sentinel-1 orbit information, which enables achieving a co-registration accuracy sufficient to preserve coherence^4^ without applying any additional refinement steps; note that due to the steep azimuth spectrum ramp across each burst acquired in Sentinel-1 TOPS mode, more stringent co-registration requirements apply when intending to use interferometric phase to estimate line-of sight displacements^41^.

**Fig. 5:**
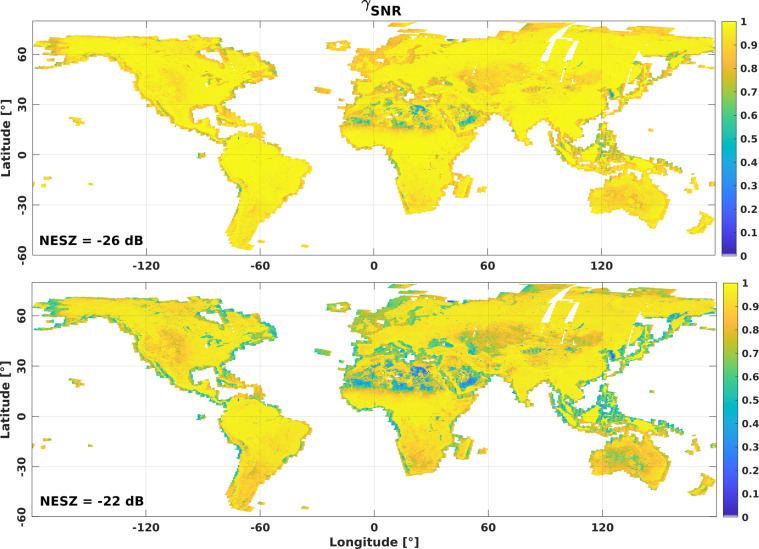
γ_noise_ simulated with the VV polarization backscatter composite for the period June, July, and August assuming an NESZ of –26 dB (top) and –22 dB (bottom).

According to Eq. (2), the coherence of repeat-pass SAR observations is determined by the signal-to-noise ratio, the loss of coherence associated with non-zero baselines, and temporal decorrelation. In areas where backscatter, *σ*^0^, is close to the noise equivalent sigma zero, NESZ, of the sensor, the low signal-to-noise ratio introduces a decorrelation contribution, *γ*_*noise*_:

4 $${\gamma }_{noise}=\frac{1}{\sqrt{1+NES{Z}_{1}/{\sigma }_{1}^{0}}\sqrt{1+NES{Z}_{2}/{\sigma }_{2}^{0}}}$$

where the indices denote the two SLCs used to form the interferogram. In the case of Sentinel-1 IW mode acquisitions at co-polarization, the NESZ presents variations in range across each burst and each of the three sub-swaths with local minima of about –30 dB and maxima of –22 dB. Based on the VV and HH polarization backscatter mosaics, we simulated the expected contribution of *γ*_*noise*_ in the coherence products with Eq. 4 assuming (i) an average NESZ of –26 dB, and (ii) a maximum NESZ of -22 dB. Figure 5 demonstrates that *γ*_*noise*_ generally presents a minor decorrelation contribution for most of the Earth’s land surface. A low signal-to-noise ratio affects the coherence primarily in arid and semi-arid regions. *γ*_*noise*_ may reach values of 0.5 or lower primarily in sandy parts of the Sahara Desert in Northern Africa, the Rub al-Chali Desert in Saudi Arabia, or the deserts in Central Australia. Somewhat dependent on the season, *γ*_*noise*_ is generally of the order of 0.8 to 0.9 across the semi-arid regions of North America, Central Asia, Australia, Southern Africa, and South America. In the Arctic regions, where Sentinel-1 acquires data primarily in HH polarization, *γ*_*noise*_ is generally close to 1. Only in summer, when melting snow/ice causes pronounced backscatter drops of 15 dB or more, does *γ*_*noise*_ become a relevant factor locally, e.g., along the coasts of Greenland.

**Fig. 6:**
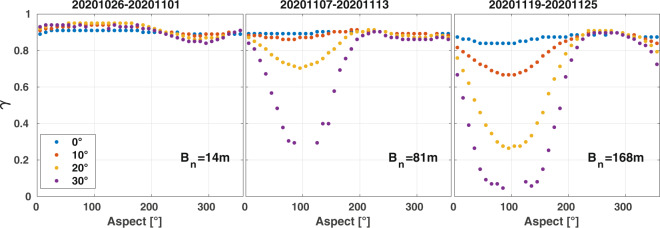
Sentinel-1 6-days repeat-pass coherence acquired over steep terrain in the Atacama Desert (Lon: 68–69°W, 24–25° S) with perpendicular baselines of 14, 81, and 168 m as function of terrain orientation and slope angles.

In the case of non-zero baselines, the two SAR acquisitions that are used to calculate the interferogram measure different parts of the ground reflectivity spectrum. This relative spectral shift, which results in a loss of coherence when unaccounted for, may be described with^42^:

5 $$\Delta f=\frac{{f}_{0}{B}_{n}}{2R{\rm{\tan }}\left(\theta -\xi \right)}$$

where *f*_0_ denotes the carrier frequency, *B*_*n*_ the perpendicular baseline, *R* the sensor-target distance, *θ* the look angle, and *ξ* the local terrain slope in range. If the baseline does not exceed the critical baseline, i.e., when *Δf* exceeds the bandwidth (~50 MHz in the case of Sentinel-1 IW), coherence can be recovered over flat terrain by applying common band filtering. On average, the perpendicular baselines were of the order of 50 m (Table 7) and seldomly exceeded the range of 150 to 200 m and were thus far below the critical baseline (~5 km). Nonetheless, coherence images calculated from interferometric image pairs with non-zero baseline do present residual decorrelation in areas of steep terrain due to residual non-common band fractions. Figure 6 illustrates that for baselines shorter than 100 m, decorrelation is mostly limited to the steepest slopes > 30° facing the sensor. In the case of baselines above 100 m, decorrelation increasingly becomes noticeable also for slopes facing the sensor of 10° to 20°. With respect to the seasonal coherence composites, which have been produced with the median of multiple coherences acquired at a given repeat interval, it is remarked that topographic effects are minor since at each location the median baselines were of the order of 50 m so that occasional coherences acquired with baselines longer than 100 m had a limited effect on the final seasonal median coherence composites.

**Table 7:** Statistical distribution of perpendicular baselines of all Sentinel-1 6- to 48-days repeat-pass interferograms.

Repeat interval [days]	Number of computed interferograms (total 7,819,631)	Perpendicular baselines [metres]			
		25^th^ percentile	50^th^ percentile	75^th^ percentile	100^th^ percentile
6	575,072	29	60	100	257
12	1,781,327	18	39	66	186
18	555,680	24	50	79	195
24	1,707,555	27	55	93	226
36	1,633,839	26	55	98	241
48	1,565,258	24	49	81	266

**Fig. 7:**
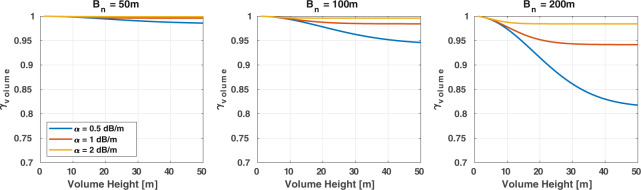
Volume decorrelation as a function of volume height, length of the perpendicular baseline, and two-way attenuation coefficient.

Another baseline dependent decorrelation term becomes relevant in the case of volume scattering from vertically distributed scatterers within a resolution cell. The effect of volume decorrelation at C-band as well as other frequencies has mostly been investigated for forests. Yet, volume decorrelation may also be relevant in other areas such as glaciers/ice sheets^28^. Experiences gathered so far based on the 1-day repeat-pass ERS-1/2 tandem mission or Sentinel-1 suggested that volume decorrelation at C-band is minor for the range of baselines at which Sentinel-1 acquires repeat-pass imagery^23,43^. When assuming an exponential decrease of the scattering power within the canopy, volume decorrelation may be modelled with^14,15^

6 $${\gamma }_{volume}=\frac{\alpha }{\alpha -j\frac{4\pi {B}_{n}}{R\lambda sin\theta }}\cdot \frac{{e}^{-j\frac{4\pi {B}_{n}}{R\lambda sin\theta }h}-{e}^{-\alpha h}}{1-{e}^{-\alpha h}}$$

where *α* is the two-way attenuation coefficient, *λ* the wavelength, and *h* the height of the volume/forest canopy. The simulation of *γ*_*volume*_ as a function of height, baseline, and signal attenuation in Fig. 7 confirms that, given the range of baselines at which Sentinel-1 acquires repeat-pass imagery, volume decorrelation hardly affects the coherence observed over forest. Only in the case of baselines» 100 m, large tree heights, and a signal attenuation of less than 1 dB/m (a potential range when the imagery was acquired, for instance, under long-term frozen conditions in the boreal zone^43^) may volume decorrelation become significant.

Given the limited, only regional relevance of *γ*_*noise*_ and *γ*_*volume*_ we may therefore conclude that temporal decorrelation presents the dominant decorrelation factor determining the level of coherence depicted in the seasonal coherence composites and the coherence decay modelling results. Nonetheless, contributions from other decorrelation mechanisms may be relevant locally when interpreting the coherence products, e.g., over deserts or steep terrain. Consideration of supporting data sets such as the backscatter and local incidence angle maps provided in the global data set is advised.

**Fig. 8:**
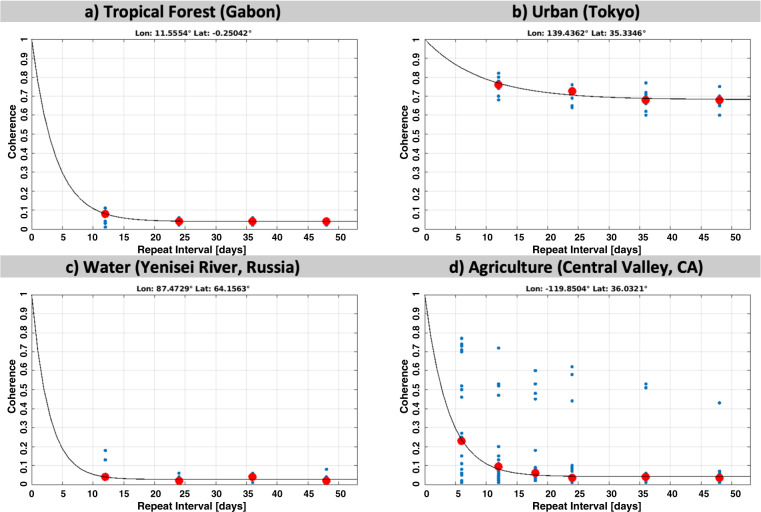
Fit of the coherence decay model in Eq. (3) to observations of the coherence at different repeat intervals. The blue dots indicate individual coherence observations, red dots the median coherence per repeat interval. Coherences observed between (**a**) June-August over a tropical forest site in Gabon (*ρ*_*∞*_ = *0.03*, *τ* = 3.98), (**b**) June-August over Tokyo, Japan (*ρ*_*∞*_ = *0.65*, *τ* = 11.3), (**c**) March-May over Yenisei River, Russia (*ρ*_*∞*_* = 0.03*, *τ* = 2.78), (**d**) June-August over an agricultural site in California, USA (*ρ*_*∞*_ = *0.03*, *τ* = 3.73).

Figure 8 illustrates a few examples of fitting the model in Eq. (3) to coherence observations (VV polarization) with either 6-, 12-, 18-, 24-, 36-, and 48-days repeat intervals (Fig. 8d) or 12-, 24-, 36-, 48-days intervals (Fig. 8a,b,c) in a given season. In most cases, we find the exponential model to allow for a reasonable description of the coherence decay with increasing repeat interval. In particular, in the case of croplands (Fig. 8d) with complex decorrelation patterns associated with field work, irrigation, and crop growth, we find the relationship between coherence and repeat interval often to be weak and the non-linear least-squares regression may even result in non-physical solutions for the model parameters, i.e., negative estimates for *ρ*_*∞*_. In such cases, we therefore adopted the Trust Region Reflective regression^39^ to allow for constraining the potential range of parameter estimates.

All backscatter, coherence, and coherence decay model parameter mosaics were visually inspected for artifacts in radiometry or geometric mismatch. No processing related artifacts were detected when zooming across the global data sets. Nevertheless, we would like to point out that the mosaics, some of which are shown in Fig. 4, do present seams associated primarily with limited data availability locally. In Northern Russia, for instance, where Sentinel-1 does not acquire data consistently in every cycle (Fig. 1), the seasonal lack of imagery or reduced data coverage did result in radiometric/coherence imbalances between adjacent orbits in the seasonal backscatter and coherence composites. Finally, users of the backscatter mosaics are advised to consider the local incidence angle maps provided with the data. The mosaics may present a dependence of the *γ*^0^ backscatter on the local incidence angle, in particular over non-vegetated terrain where the conversion from *σ*^*0*^ to *γ*^0^ does not suffice to compensate for/minimize such dependence^44^.

## Usage Notes

This data set is available for unlimited use under the Creative Commons License 4.0. We encourage users to develop cloud-based solutions to interact with the data set, e.g., via the AWS resource of the data set. Notebook examples are provided. The suite of GDAL tools is efficient in subsetting the data sets via VRTs. A python tool *global_coherence_mosaic_tool.py* to access data with mosaicking of manageable subsets is provided in the available open source code (https://github.com/EarthBigData/openSAR/blob/EBDopenSAR-2021-11-11/code/global_coherence/README.md). The tools allow users to specify geographic subsets and selections of metrics to download and generate VRT or GeoTIFFs based on tile lists or ranges of latitude and longitude. Users are advised to estimate download volume size before choosing subsets. The entire global data set for all metrics has a volume of about 2.2 Terabytes. The provided tool also allows for the generation of http access URLs as text files which can be input to downloading large amounts of data via commonly available download tools like *wget* or *curl*. The global VRTs and mosaics can directly be visualized in geographic information systems and has been tested with QGIS (https://qgis.org) and the AWS resource.

## Data Availability

The interferometric processing of Sentinel-1 data was performed with processing scripts using the commercial software developed by GAMMA Remote Sensing (v20201216). Usage of the software is subject to licensing (https://www.gamma-rs.ch/software). A open code repository for the core code developed for processing, data access, and visualization is available at the Earth Big Data LLC *openSAR* github repository^45^. Relevant code for this data set is available in the *global_coherence* folders in the *code* and *notebooks* sections at this repository. A python tool to mosaic and subset data from the tiles is available at: https://github.com/EarthBigData/openSAR/blob/master/code/global_coherence/global_coherence_mosaic_tool.py.
